# Characterizing the Baseline Regional Biphasic Mechanical Properties of Cervical Intervertebral Discs

**DOI:** 10.1007/s10439-025-03759-2

**Published:** 2025-05-21

**Authors:** Nathan Buchweitz, Yi Sun, Joshua Kelley, Sarah Cisewski Porto, Shangping Wang, Charles A. Reitman, Hai Yao, Yongren Wu

**Affiliations:** 1 Department of Bioengineering, Clemson University, Clemson, SC USA; 2 Department of Orthopaedics, The 2nd Affiliated Hospital of Harbin Medical University, Harbin, China; 3 School of Health Sciences, College of Charleston, Charleston, SC USA; 4 Department of Orthopaedics and Physical Medicine, Medical University of South Carolina, Charleston, SC USA

**Keywords:** Cervical spine, Intervertebral disc, Biphasic properties, Finite element analysis

## Abstract

**Purpose:**

While the regional viscoelastic biomechanical properties of lumbar intervertebral disc tissues are well documented, equivalent tissue-level characterizations for human cervical discs remain unexplored. This study aimed to quantify biphasic mechanical properties of the nucleus pulposus (NP), annulus fibrosus (AF), and cartilaginous endplate (CEP) in cervical discs.

**Methods:**

A previously established confined compression testing technique was used to measure swelling pressure, equilibrium aggregate modulus, and hydraulic permeability in cervical NP, AF, and CEP tissues. Specimen-specific porosity was also assessed and correlated with these properties. A finite element model was used to simulate unconfined compression.

**Results:**

Swelling pressure (154.50 ± 89.47 kPa) and aggregate modulus (0.677 ± 0.671 MPa) were significantly higher in the CEP compared to the NP (*p* = 0.0308 and *p* = 0.0227, respectively) or AF (*p* = 0.0338 for aggregate modulus), with no significant differences observed between NP and AF. Permeability did not differ significantly among regions. Porosity showed negative correlations with both swelling pressure (*r* = − 0.55, *p* = 0.0006) and aggregate modulus (*r* = − 0.53, *p* = 0.001). Finite element analysis revealed a relatively uniform von Mises stress distribution between NP and AF, with higher magnitudes concentrated in the CEP.

**Conclusion:**

Cervical NP and AF exhibit relatively homogeneous biomechanical properties, whereas the CEP is found to have greater stiffness and swelling pressure. These findings indicate unique tissue-level adaptations in cervical discs to support greater mobility. These data could also inform future studies investigating region-specific degeneration and aging effects on cartilaginous tissue function in cervical discs and enhance the representation of viscoelasticity in computational modeling of the IVD.

**Supplementary Information:**

The online version contains supplementary material available at 10.1007/s10439-025-03759-2.

## Introduction

Intervertebral discs (IVDs) are avascular cartilaginous tissues situated between vertebrae, which serve essential roles in weight-bearing, mechanical shock absorption, and constraining three-dimensional spinal movements [1–3]. IVDs share a common structural organization throughout the spine, comprising the nucleus pulposus (NP) at the core, the annulus fibrosus (AF) as a peripheral boundary, and cartilaginous endplates (CEP) interfaced superiorly and inferiorly with vertebrae [2–4]. Despite this general similarity, variations in disc size, kinematics, internal tissue structure, and biochemical composition are evident along the spine, reflecting adaptations to unique biomechanical environments [1, 4–6]. Cervical IVDs, which are essential for facilitating head mobility and supporting sensory functions such as hearing, vision, and olfaction, differ markedly from lumbar IVDs, which are regarded for their weight-bearing capacity [1, 7]. Cervical discs allow greater ranges of lateral flexion and axial rotation compared with their lumbar counterparts [8, 9]. Structurally, cervical discs are more fibrocartilaginous, particularly in the NP [10]. Unlike lumbar discs, the vertebral bone interface exhibits a more saddle-shaped morphology, and the cervical AF does not always fully encapsulate the NP posteriorly [1, 8, 11]. These features, which become more apparent in cranial segments, likely contribute to increased kinematic freedom [1, 8, 11]. Biochemically, cervical discs contain higher collagen levels and exhibit a more homogeneous distribution of proteoglycans and water within their extracellular matrix compared with lumbar discs [6, 12].

While cervical discs’ structural and compositional distinctions are well documented, their tissue-level mechanical properties remain poorly characterized compared to intact motion segments [4, 7]. In lumbar discs, the soft tissue exhibits viscoelastic behavior in compression, due in part to fluid flow and redistribution within tissue during loading [2]. This phenomenon is especially evident in tissues like the NP, which feature a high water content and loosely organized matrix [13]. A biphasic material framework can be employed to gain insights into the intrinsic solid–fluid interactions in various cartilaginous tissues under compression [14–19]. Biphasic mechanical parameters including aggregate modulus and permeability have been regionally quantified in human lumbar discs [17, 20–25]. However, similar quantification for cervical AF, NP, and CEP tissues is currently lacking. This knowledge gap limits a more comprehensive understanding of the regional mechanical interactions within cervical discs, and impedes the development of accurate biomechanical models essential for investigating head and neck injuries [26–31].

The present study aims to regionally quantify aggregate modulus, hydraulic permeability, and swelling pressure in cervical discs using an established confined compression creep testing protocol [24, 25, 32]. We hypothesized that the AF and NP would exhibit relatively uniform mechanical properties, particularly in aggregate modulus, due to their more homogeneous water content [6]. The CEP was expected to demonstrate higher swelling pressure and aggregate modulus, consistent with its role as a protective mechanical interface at the disc–vertebra interface [25, 32, 33]. Correlation analyses were conducted to examine the relationship between tissue porosity and biphasic properties across regions. Additionally, a finite element model was developed to simulate and visualize the biphasic mechanical response of cervical discs under unconfined compression. It was hypothesized that the AF and NP would display relatively homogeneous stress distributions, with peak stresses localized in the CEP consistent with the regional mechanical properties. This study provides foundational data for cervical disc biomechanical modeling and offers insights that may support future research on cervical IVD degeneration and aging.

## Materials and Methods

### Sample Preparation

Five male cadaveric cervical spines (mean age 59 ± 13 years) were obtained in a fresh-frozen state from Science Care (Phoenix, AZ), with approval from the Medical University of South Carolina. Discs were first isolated by cutting through adjacent vertebrae using a surgical bone saw. Harvested segments were then individually wrapped in plastic, and gauze soaked in phosphate-buffered saline (1xPBS, pH 7.4) to prevent dehydration [24]. To mitigate impacts on tissue mechanical properties, segments were stored frozen at − 20 °C for up to 48 hours [34–37]. AF, NP, and CEP samples were prepared immediately prior to biomechanical testing. Each disc region was graded for gross morphological defects, including fissures and macro-calcification, using the scale developed by Thompson, et al. [38]. Grading was performed by a clinical collaborator (C.R.). Only grade II and III specimens were biomechanically tested. Two of the donors featured moderate to severe degeneration (grades IV–V) in some of the discs and were reserved for future studies. The remaining donors supplied discs from various levels within the cervical spine (C2–C3, C3–C4, C4–C5, and C6–C7 harvested from one donor, C2–C3, C3–C4, and C4–C5 from another, and C2–C3, C4–C5, C6–C7, and C7–T1 from the remaining donor).

**Fig. 1:**
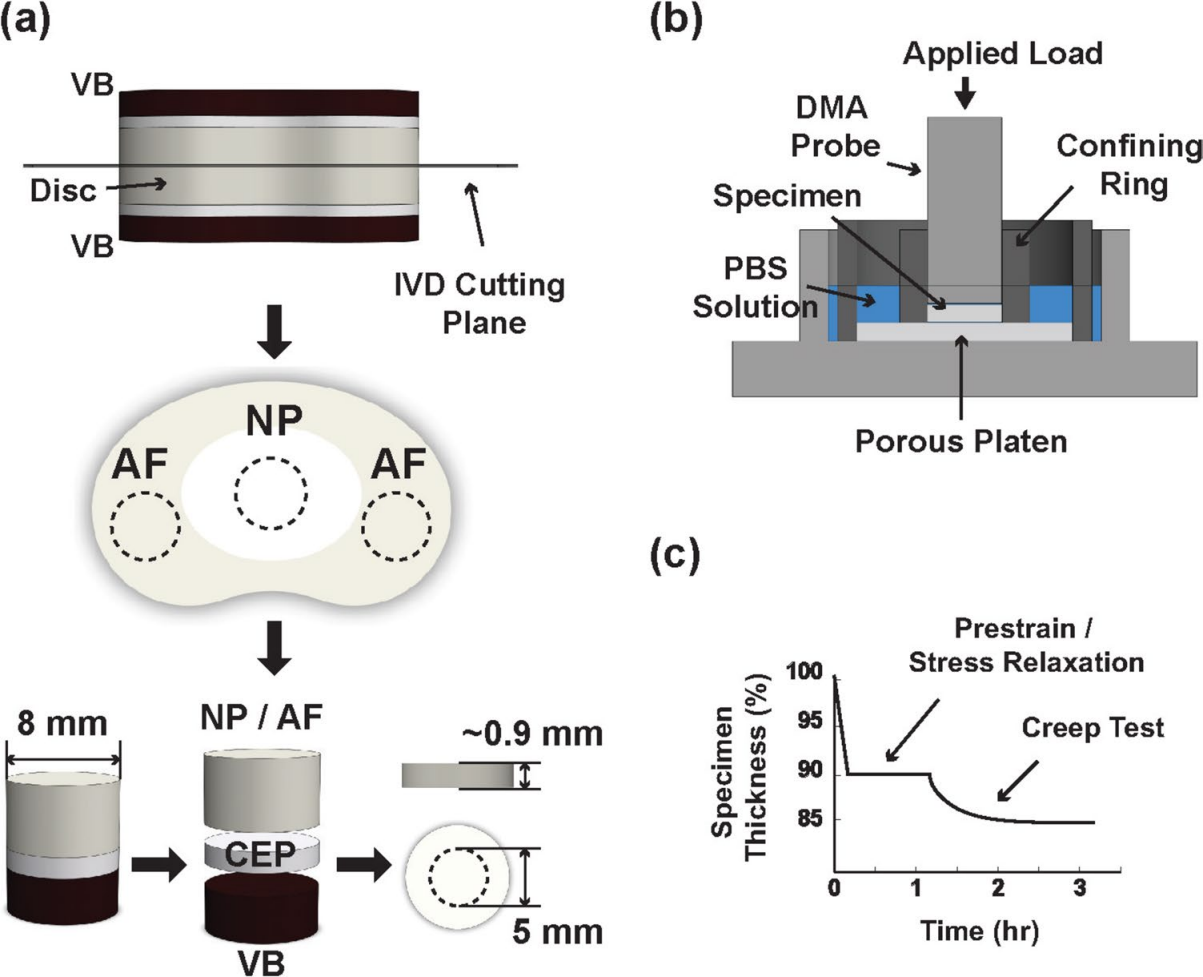
Schematic of the **a** sample preparation, **b** confined compression testing apparatus, and **c** biomechanical testing protocol

To prepare the AF, NP, and CEP samples for biomechanical testing, discs were first opened through the mid-plane using a sterile surgical scalpel. An 8-mm corneal trephine was then used to extract osteochondral plugs from the disc halves, containing vertebral bone as well as the intact CEP and either AF or NP tissue (Fig. 1a). AF and NP samples were sectioned to approximately 1 mm thickness, while CEP samples were sectioned to 0.9 mm using a freezing-stage microtome (M2400, Leica Microsystems GmbH, Wetzlar, Germany). For CEP samples, vertebral bone was removed first, followed by the NP or AF tissue, where a 0.9-mm-thick plastic spacer was used as a cutting reference. Finally, a 5 mm trephine was applied to shape the samples with a cylinder geometry suitable for testing. Prepared sample thicknesses were assessed using a current-sensing digital micrometer [24, 39], averaging 1.17 ± 0.35 mm for disc tissues and 0.92 ± 0.21 mm for CEP.

### Biomechanical Testing Procedure

Confined compression experiments were conducted following established protocols using a Thermal Advantage Dynamic Mechanical Analyzer (TA-Q800 DMA, New Castle, DE) equipped with a custom-designed confining chamber clamp (Fig. 1b) [24, 25, 40]. The DMA measured force and displacement with 1 mN and 1 µm precisions, respectively. Regionally prepared IVD samples were inserted into the test chamber, laterally confined by 5-mm-diameter walls, and compressed axially between a porous platen (20 µm pore size) at the base and the DMA displacement probe at the top surface (Fig. 1b). Samples were oriented with the side facing the vertebral bone positioned against the porous platen.

The testing protocol included a stress relaxation phase followed by a creep phase (Fig. 1c). Before stress relaxation, samples were immersed in PBS and allowed to swell freely in the axial direction. Periodically (in ~ 15-min intervals), the PBS was replenished in the testing chamber. Samples were then compressed to 10% strain relative to their thickness, as measured during preparation with a digital micrometer. This ensured sample contact with the DMA probe and interdigitation with the porous platen. After approximately one hour of stress relaxation, the equilibrium load was recorded and was divided by the cross-sectional area of the sample (19.63 mm^2^) to calculate swelling pressure. The creep phase followed, where an additional load was applied over a 5-minute ramp, equivalent to 20% of the relaxation equilibrium load. This produced creep strains of approximately 3–5% after two hours. The linear biphasic theory was used to curve-fit the creep strain data, yielding measurements of hydraulic permeability and equilibrium aggregate modulus (see Online Resource 1, Supplemental Fig. 1 for experimental data traces) [14].

### Regional Porosity Measurement

A buoyancy weighing technique was used to measure the water volume fraction (porosity) in biomechanically tested samples. Samples were first weighed in air (${W}_{wet}$) using an analytical balance with 100 µg precision. After biomechanical testing, samples were again weighed while submerged in PBS (${W}_{PBS}$) using a density determination kit (Sartorius, Germany). Following one week of lyophilization, the dry weight (${W}_{dry}$) was recorded. Porosity (${\phi }^{w}$) was calculated based on a previously established method using Eq. 1, where $\frac{{\rho }_{PBS}}{{\rho }_{w}}$ = 1.005 is the density ratio of PBS to water [24, 41, 42]:

1 $$\phi^{{\text{w}}} = \frac{{W_{{{\text{wet}}}} - W_{{{\text{dry}}}} }}{{W_{{{\text{wet}}}} - W_{{{\text{PBS}}}} }}*\frac{{\rho_{{{\text{PBS}}}} }}{{\rho_{{\text{w}}} }}$$

### Multiphoton Microscopy

The collagen fiber structure was examined in the NP, AF, and CEP regions from a representative disc at the C6–C7 level. An 8 × 6 × 4 mm block of tissue was sectioned in the sagittal plane for this purpose. Each region was imaged over a 2 × 2 mm area using a custom-built microscope equipped with a Chameleon Ultra laser (Coherent Corp., Saxonburg, PA) and operated with an excitation wavelength of 740 nm [43, 44]. A second harmonic generation (SHG) emitted signal was collected in the 334–406 nm range, while extracellular matrix structures and cell morphology were captured in the 400–484 nm and 537–677 nm fluorescent emission ranges [43, 44]. Image stacks (512 × 512 pixels, 547 × 547 μm^2^) were acquired to a depth of 50 μm in 2 μm steps. The fibers in the AF region were purposefully orientated perpendicular to the imaging plane for ease of visualization. Composite images were assembled in ImageJ and then stitched using Adobe Photoshop 2024 (Adobe, San Jose, CA) [45].

### Cervical Disc Finite Element Model

**Fig. 2:**
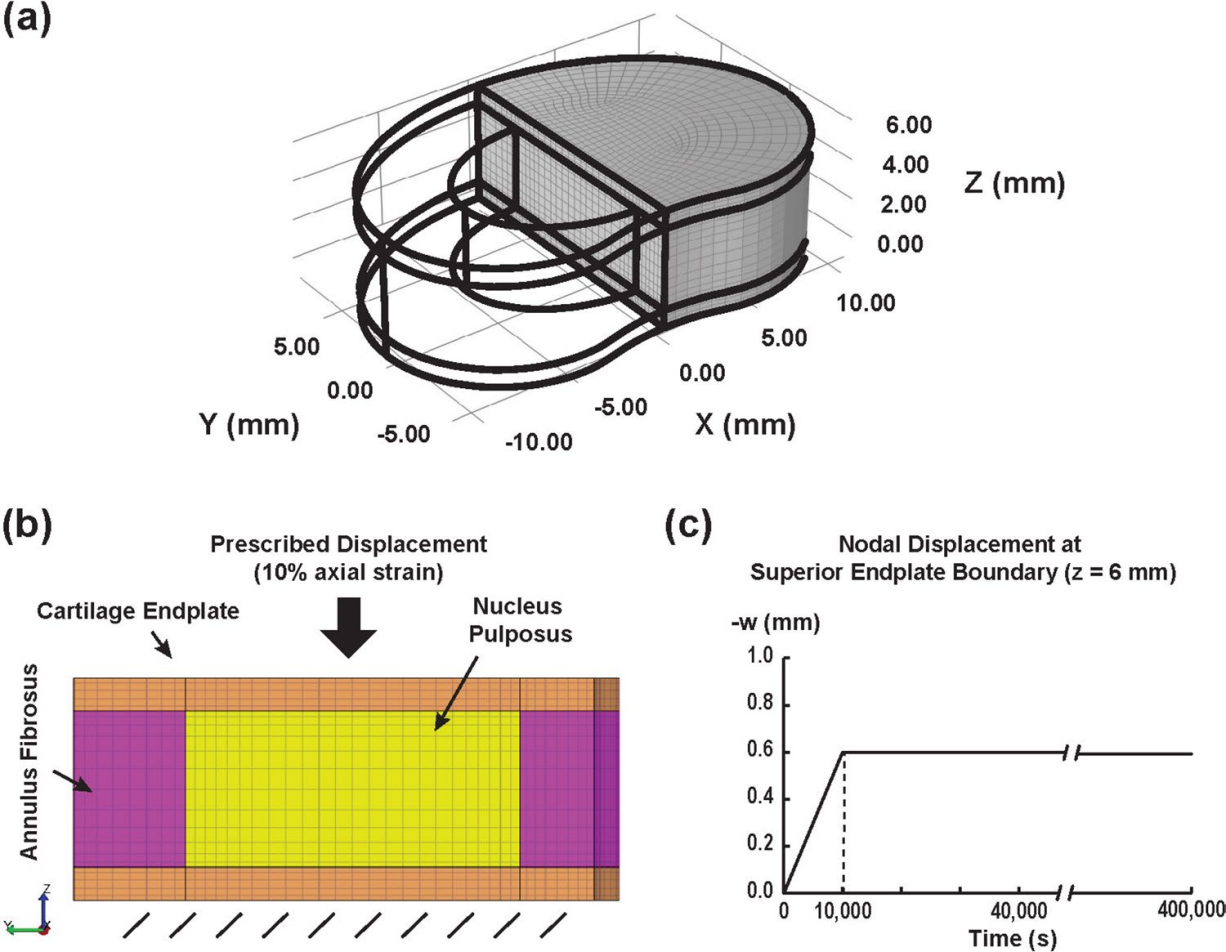
Cervical disc finite element model; **a** mesh, **b** material regions and test configuration, and **c** simulated protocol for unconfined compression

A finite element model was developed to examine the mechanical response of a cervical intervertebral disc under static unconfined compression, incorporating the regional biphasic mechanical properties measured in this study. The analysis focused on the effective (von Mises) stress distribution, fluid pressure, and fluid flux within the disc. The disc geometry, characterized by its bean shape and three distinct soft-tissue domains (AF, NP, and CEP), was derived from transverse-plane dissection images of a cadaveric C6–C7 segment (schematic in Online Resource 1, Supplemental Fig. 2). Endplate thickness was 0.9 mm, based on average sample thickness and microscopy images, while disc height was set to 6 mm, based on upper range estimates from morphometric literature [46]. For computational efficiency, symmetry was leveraged by modeling only one-half of the disc (Fig. 2a). Exposed IVD surfaces (including those of the endplates) were assigned free-draining boundary conditions (zero fluid pressure). The bottom surface of the inferior endplate was fixed in the axial direction, and a 10% axial compressive strain was applied to the top surface of the superior endplate for a period of 10,000 s (Fig. 2b, c) [47].

A structured mesh consisting of 15,617 nodes and 14,036 trilinear hexahedral (8-node) elements was generated using COMSOL Multiphysics (Version 5.6, COMSOL Inc., Burlington, MA), and exported for analysis within the open-source finite element software FEBio (Fig. 2a, b) [48]. The model incorporated biphasic materials, with an isotropic elastic solid phase and isotropic permeability assigned to each IVD region. The Poisson ratio was kept consistent across regions (ν = 0.2) at their approximate average reported in prior IVD literature [20]. Young’s modulus (E) was calculated from the equilibrium aggregate modulus (H_A_) according to the following relationship (Eq. 2):

2 $$E = \frac{{H_{{\text{A}}} \left( {1 + v} \right)\left( {1 - 2v} \right)}}{{\left( {1 - v} \right)}}$$

Permeability values were derived from regional biomechanical testing data. All material parameters are summarized in Table 1. Simulations incorporating a biphasic-swelling model were performed to extend the analysis, accounting for deformation-dependent osmotic pressure effects within the disc [20]. This was achieved by modeling Donnan equilibrium swelling behavior as part of a solid-phase mixture material in FEBio. The swelling model incorporated a regional fixed charge density term (Table 1) and a simulated external saline environment (e.g., sodium chloride) with a bath concentration of c^*^ = 150 mM [47]. This model was allowed to equilibrate completely for all mechanical outcomes of interest before proceeding with the compressive ramp.

**Table 1:** Summary of finite element model material parameters used for AF, NP, and CEP tissues

Parameter	NP	AF	CEP
*E* (MPa)	0.39	0.52	1.07
*v*	0.2	0.2	0.2
*H*_A_ (MPa)	0.44	0.57	1.19
*k*_0_ (10^−4^ mm^4^/N/s)	2.96	2.75	1.93
${\phi }_{0}^{s}$	0.127	0.183	0.307
FCD (mM)**	182.2	135.5	286.7
c^*^ (mM)	150	150	150

*E* Young’s Modulus, *v* Poisson’s ratio, *H*_A_ aggregate modulus, *k*_0_ isotropic permeability

${\phi }_{0}^{s}$: solid volume fraction, *FCD* fixed charge density^**^ (swelling model only), c^*^ bath osmolarity (swelling model only)

^**^FCD regional values borrowed from a related cervical IVD study[70]

### Statistical Analysis

Linear mixed models were used to examine the dependence of measured biomechanical properties (swelling pressure, aggregate modulus, permeability) on the IVD region. These models were fit using R software [49, 50], incorporating a random effect for spine donors as determined by likelihood-based model selection. Model-based averages and 95% confidence intervals for each IVD region are presented, with corresponding mean ± SD values provided in Table 2. Regional differences in IVD properties were evaluated using one-way ANOVA and pairwise comparisons between AF, NP, and CEP regions using a generalized t-test procedure with Bonferroni–Holm adjusted p-values [51, 52]. Additionally, Pearson correlations were calculated between each biomechanical property and tissue porosity, pooled across all regions. Statistical significance was set at *p* < 0.05 for all tests.

**Table 2:** Comparison of biphasic mechanical properties measured from human intervertebral disc tissues

Tissue	Method	Aggregate modulus (MPa)	Permeability (10^−16^ m^4^/Ns)	References
Cervical AF	Confined compression	0.57 ± 0.48	2.75 ± 2.29	This study
Cervical NP	Confined compression	0.44 ± 0.44	2.96 ± 2.51	This study
Cervical CEP	Confined compression	1.19 ± 0.98	1.93 ± 1.74	This study
Lumbar AF	Confined compression	0.03 ± 0.02	64 ± 76	[20]
Lumbar AF	Confined compression	0.56 ± 0.21	1.8 ± 0.7	[21]
Lumbar NP	Confined compression	0.10 ± 0.07	5.5 ± 7.8	[20]
Lumbar NP	Confined compression	1.01 ± 0.43	9.0 ± 4.3	[17]
Lumbar CEP	Confined compression	1.86 ± 1.24	1.4 ± 1.0	[25]
Lumbar CEP	Confined compression	~ 0.26	~ 5.6	[22]
Lumbar CEP	Permeameter	−	(1.19 ± 1.64) × 10^6^	[23]

## Results

### Regionally Mapped Biomechanical Properties

**Fig. 3:**
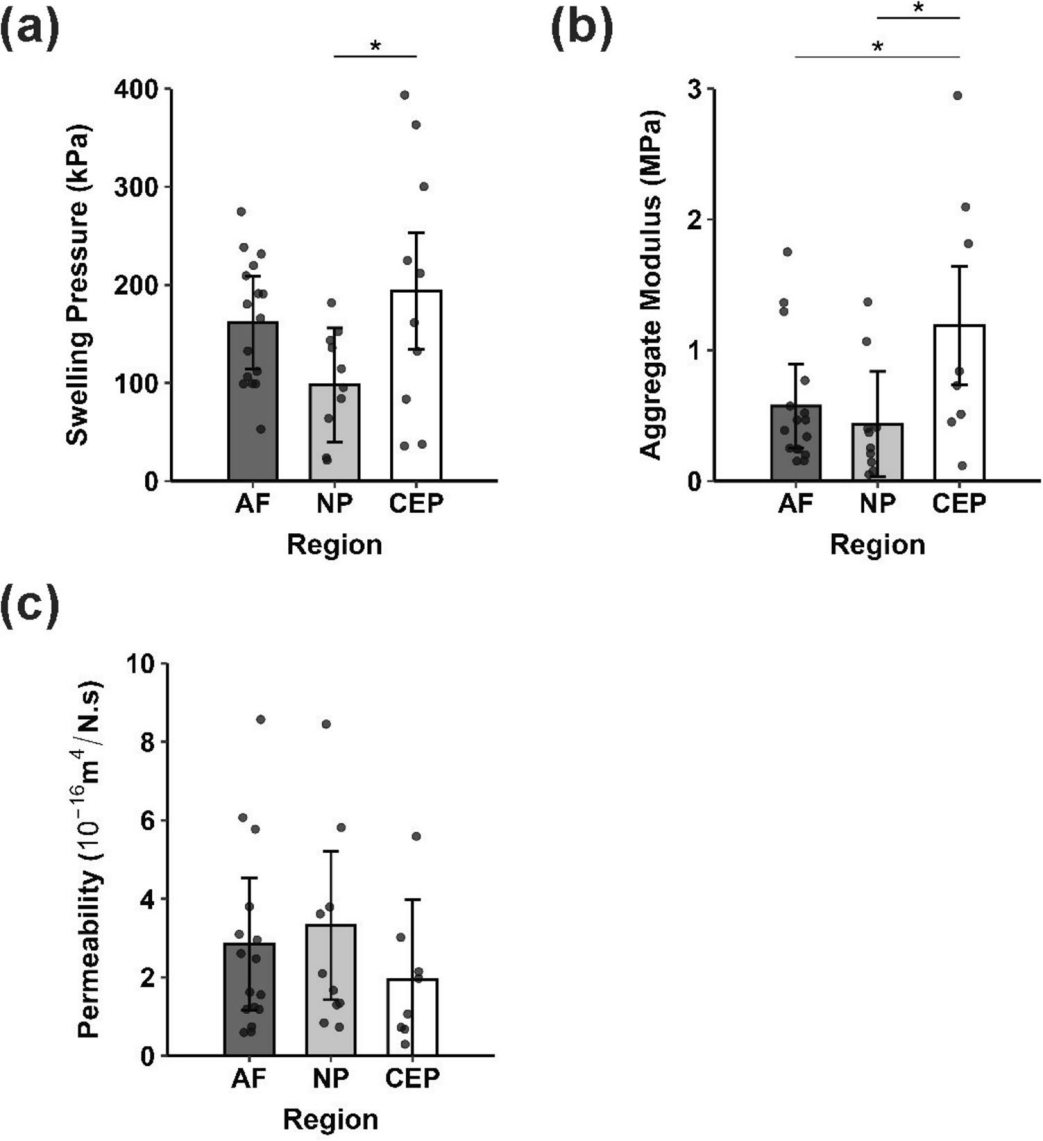
Regional biomechanical properties of cervical IVD tissues as measured by confined compression; **a** swelling pressure, **b** aggregate modulus, and **c** hydraulic permeability. Sample sizes per region were AF: n = 16, NP: n = 10, CEP: n = 10 for **a** or n = 8 for **b** and **c** due to sample damage during the creep testing phase. *p < 0.05

Swelling pressure was found to be regionally heterogeneous (154.50 [95% CI 124.23, 184.77] kPa, *p* = 0.0447) in the cervical discs. Specifically, a higher swelling pressure was determined in the CEP region (193.57 [134.33, 252.80] kPa) compared to the NP region (97.86 [39.71, 156.00] kPa, *p* = 0.0308). However, no significant differences in swelling pressure were found between the AF (161.37 [113.89, 208.86] kPa) and NP or between the AF and CEP (Fig. 3a). Aggregate modulus also exhibited regional heterogeneity (0.68 [0.44, 0.91] MPa, *p* = 0.0286). The CEP region had a greater aggregate modulus (1.19 [0.74, 1.64] MPa) compared with both the NP (0.44 [0.03, 0.84] MPa, *p* = 0.0227), and the AF (0.57 [0.26, 0.89] MPa, *p* = 0.0338). No differences were observed between the AF and NP (Fig. 3b). Permeability was not seen to vary significantly by region (AF: 2.854 [1.171, 4.537] 10^−16^ m^4^/N/s, NP: 3.321 [1.426, 5.215] 10^−16^ m^4^/N/s, CEP: 1.947 [0.000, 3.989] 10^−16^ m^4^/N/s, Combined: 2.623 [1.851, 3.395] 10^−16^ m^4^/N/s; *p* = 0.4449; Fig. 3c).

### Correlation of Biomechanical Properties with Tissue Porosity

**Fig. 4:**
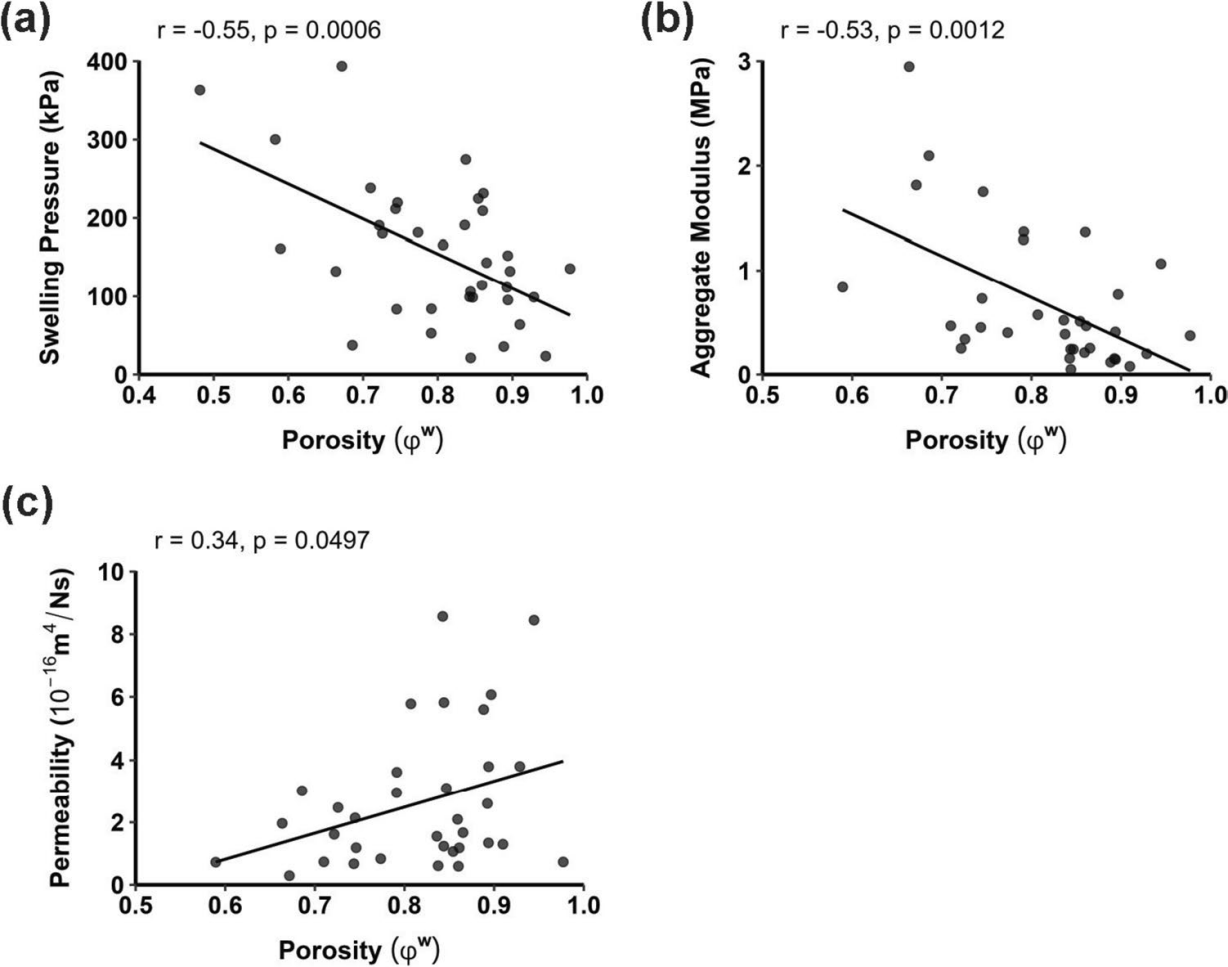
Correlation of cervical IVD tissue porosity with **a** swelling pressure, **b** aggregate modulus, and **c** hydraulic permeability. Sample sizes per region were AF: n = 16, NP: n = 10, CEP: n = 10 for **a** or n = 8 for **b** and **c** due to sample damage during the creep testing phase.

A significant negative correlation was found between swelling pressure and porosity (*r* = − 0.55, *p* = 0.0006) as well as between aggregate modulus and porosity (*r* = − 0.53, *p* = 0.0012), representing moderate associations (Fig. 4a, b). A significant positive correlation was found between permeability and tissue porosity (*r* = 0.34, *p* = 0.0497), although this trend was weak (Fig. 4c).

### Extracellular Matrix Structure

**Fig. 5:**
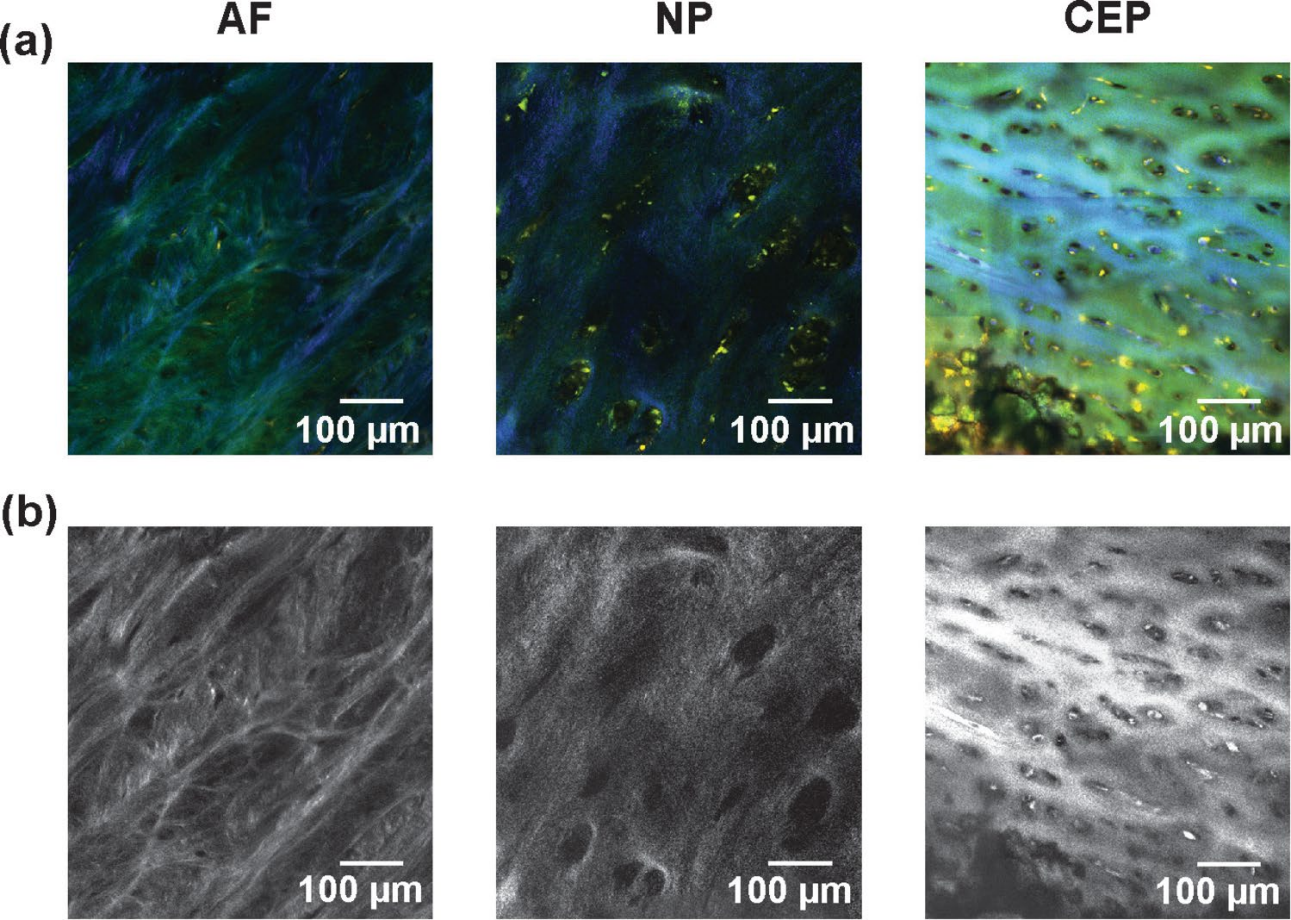
Multiphoton microscopy of representative AF, NP, and CEP regions of the cervical IVD. Images include **a** a composite of SHG signal (to visualize collagen) [blue: 420–460 nm collection range] and two-photon excited fluorescence (to observe cellular structures and other ECM components) [green: 420–460 nm range; red: 575–630 nm range] and **b** standalone SHG signal in grayscale. The NP region has visible fibrous inclusions, and does not show the typical gelatinous structure described for lumbar NP tissue [2, 55]. The CEP region, similar to lumbar CEP, is seen to have a transversely aligned matrix [25]. Scale bar = 100 μm.

Multiphoton microscopy revealed regionally distinct collagen fiber morphologies (Fig. 5a, b). In the AF, collagen fiber bundles are highly aligned and connected by smaller branching fibers. The NP is also dense with collagen, but this network appears relatively smooth in its topology, intermixed with a disordered arrangement of cavities, where cell lacunae appear. In the CEP, the cavities in the collagen matrix are denser and more regular, showing a distinctive transverse alignment with the bone interface. They become progressively more rounded further from the bone.

### Simulated Compression of a Cervical Disc

**Fig. 6:**
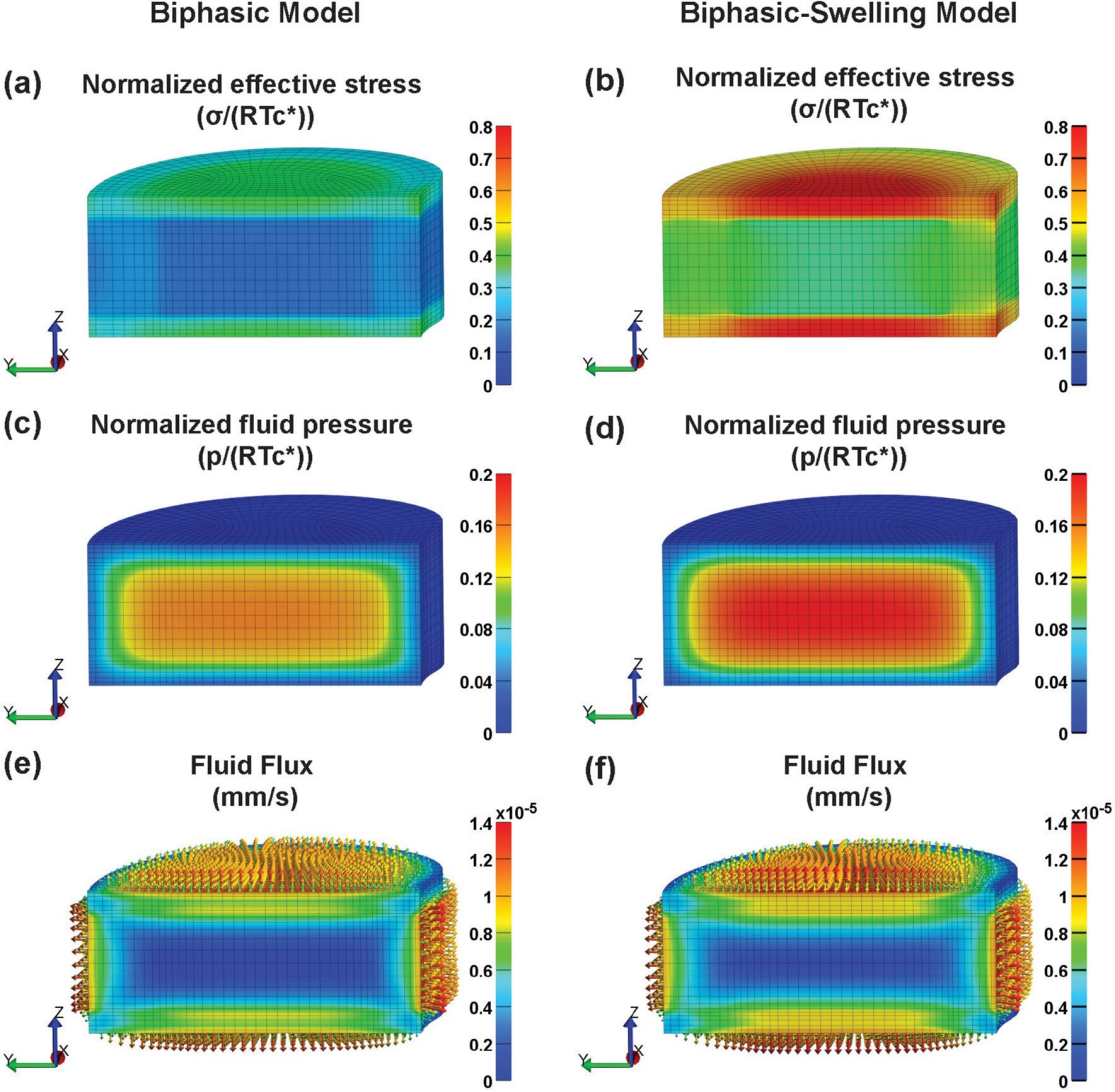
The biphasic and extended biphasic-swelling material models predictions of **a**–**b** contours of normalized effective (von Mises) stress, **c**–**d** normalized fluid pressure, and **e**–**f** fluid flux magnitude in a cervical disc (C6-C7) following a ramped 10% axial compressive strain (time point = 10,000 s).

**Fig. 7:**
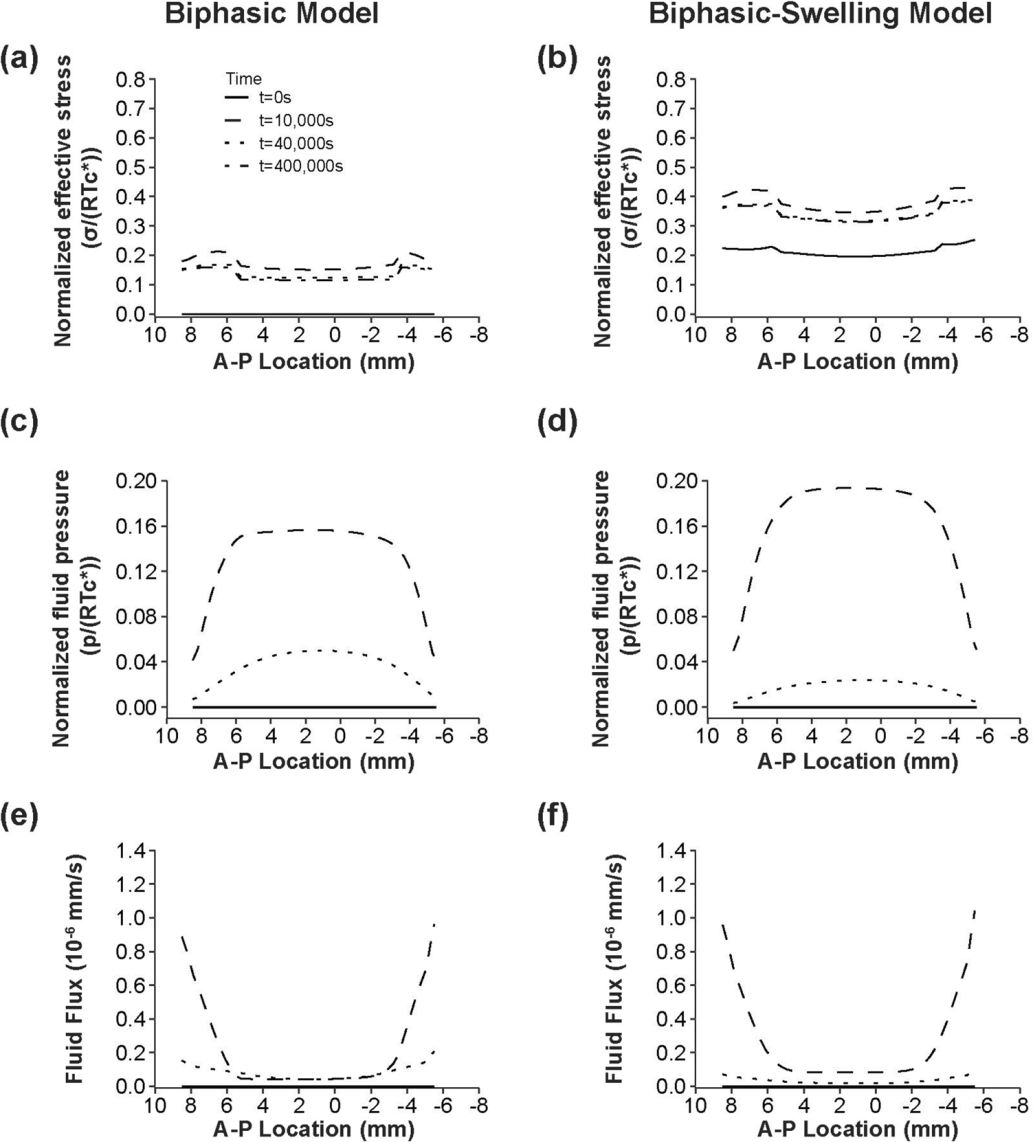
The biphasic and extended biphasic-swelling material models predictions of **a**–**b** the normalized transient response of effective (von Mises) stress, **c**–**d** normalized fluid pressure, and **e**–**f** fluid flux magnitude in the anterior–posterior (A–P) axis of the cervical disc during stress relaxation at various time points

At the end of the ramp compression phase, the effective stress distribution in the sagittal plane was relatively uniform between the NP and AF regions of the disc (Figs. 6a, b, 7a, b). This trend was consistent across both FE models, though the swelling model predicted stress magnitudes approximately twice as large as the non-swelling model (Table 3). Notably, the CEP exhibited much higher effective stress magnitudes than the NP and AF, with peak values exceeding those in the NP by twofold (Fig. 6a, b; Table 3).

**Table 3:** Finite element model peak outcomes (maximum magnitude), summarized by region and time point

Time point	Peak outcome	Biphasic model			Biphasic-swelling model		
		NP	AF	CEP	NP	AF	CEP
10,000 s (end of compression)	Effective stress (MPa)	0.063	0.087	0.146	0.144	0.190	0.294
	Fluid pressure (kPa)	59.12	60.07	38.96	72.28	72.23	45.93
	Fluid flux (10^−5^ mm/s)	0.864	1.320	1.198	0.947	1.321	1.313
	A–P Lagrange strain	0.093	0.076	0.066	0.278	0.238	0.232
	I–S Lagrange strain	− 0.167	− 0.189	− 0.124	− 0.132	− 0.177	− 0.061
400,000 s (near equilibrium)	Effective stress (MPa)	0.044	0.060	0.077	0.127	0.155	0.210
	Fluid pressure (kPa)	5.13 × 10^−4^	3.34 × 10^−4^	2.83 × 10^−4^	3.62 × 10^−6^	2.55 × 10^−6^	2.06 × 10^−6^
	Fluid flux (10^−5^ mm/s)	6.03 × 10^−6^	6.65 × 10^−6^	7.78 × 10^−6^	4.19 × 10^−8^	4.59 × 10^−8^	5.32 × 10^−8^
	A–P Lagrange strain	0.028	0.031	0.020	0.228	0.198	0.194
	I–S Lagrange strain	− 0.151	− 0.154	− 0.079	− 0.138	− 0.165	− 0.014

*A–P* Anterior–posterior axis, *I–S* Inferior–superior axis

The fluid pressure distribution displayed a gradient between the NP and AF regions, progressively diminishing as the simulation approached equilibrium (Figs. 6c, d, 7c, d; Table 3). Fluid flux was most pronounced at the peripheral boundary of the AF immediately following the compressive ramp (Fig. 6e, f; Table 3). The fluid flux gradient between the AF and NP regions exhibited an inverse pattern to that of fluid pressure (Fig. 7e, f). Although the fluid flux through the middle portion of the outer CEP boundaries was slightly lower than in the AF, it was considerably higher than at any location within the NP. As the simulation progressed, the residual flux became more prominent in the CEP compared to the AF (Table 3).

## Discussion

Aggregate modulus, swelling pressure, and permeability in cervical discs did not differ significantly between the NP and AF, suggesting greater regional homogeneity than in lumbar discs (Table 2). The aggregate modulus reflects the stiffness of the collagen matrix under equilibrium loading conditions, while swelling pressure represents the effects of the osmotic environment on interstitial fluid pressure [2, 53]. Relative homogeneity in these outcomes could suggest that cervical discs are biomechanically adapted to support multidirectional motions, such as bending and torsion. Supporting this interpretation, a prior study reported that cervical motion segments experience flexional bending moments approximately 20% of the magnitude of those in lumbar discs, along with significantly higher thresholds for bending moment failure [7, 54]. Cervical disc segments have also been seen to have a lower ultimate axial compressive strength, less than half that of lumbar discs of similar age, underscoring a reduced specialization for axial load bearing [7]. While the biphasic properties of cervical tissues presently reported are comparable in magnitude to lumbar tissues, the cervical NP featured a lower permeability and in one case a greater aggregate modulus (Table 2). Prior literature suggests that cervical IVDs tend to be more fibrotic in the NP, even as early as the second decade of life, which may explain this trend [6, 10]. Microscopy images also reveal a fibrous cervical NP structure which differs from the gel-like structure frequently described for lumbar NP (Fig. 5b) [2, 55].

The CEP exhibited distinct mechanical properties, featuring significantly greater swelling pressure and aggregate modulus than the NP and AF (Table 2). These findings support its role as a transitional mechanical barrier at the disc–vertebra interface, where the greater CEP stiffness may facilitate the transmission and distribution of compressive loads [25, 32]. Similar to the lumbar CEP, the lower permeability observed in the cervical CEP, although not statistically significant, suggests a dual function in limiting fluid convection and retaining nutrients during repetitive loading [25, 56–58]. Aggregate modulus was slightly lower and permeability slightly greater in the cervical CEP compared with lumbar CEP measurements (Table 2). It is possible that the cervical CEP interface is better supported by the underlying vertebral endplate layer, which is stiffer due to the smaller vertebral bodies, thicker cortical shells, and increased bone mineral density [7, 59–61]. Reduced endplate areas may additionally necessitate greater permeability. Microscopy images of the CEP interface from this study revealed a distinct transverse alignment of the collagen matrix near the bone, which diminishes further into the disc (Fig. 5c). This suggests a critical role for tensile and shear properties near the bone interface, which have yet to be quantified in cervical discs [25, 62].

Pooled across regions, moderate negative correlations were found between tissue porosity and both swelling pressure and aggregate modulus (Fig. 4a, b). This trend was consistent with that reported in other IVD tissues [25, 32, 63]. Less porous tissues, such as the CEP, exhibited greater stiffness, consistent with this relationship. With greater porosity, increased variability in water content may contribute to the biomechanical similarity observed between the NP and AF. Although permeability correlated weakly and positively with porosity, high variability likely explains the absence of significant regional differences (Fig. 4c).

Finite element modeling was used to visualize the likely impacts of regional biphasic mechanical properties on cervical disc stress distributions under unconfined compression. Regardless of the incorporation of swelling effects, both models revealed relatively homogeneous stress distributions in the NP and AF regions, with elevated stress concentrations localized to the CEP (Table 3). This seemed to indicate that while swelling effects were important contributors to the initial stress and fluid pressure states, they did not otherwise impact the regional biomechanical environment of cervical discs. Compared with a simulated model of a lumbar disc incorporating osmotic effects, the normalized pattern of effective stress was considerably more uniform across NP and AF regions, specifically revealing less concentrated stress in the NP–AF transition zone [47]. Beyond facilitating bending mobility, a reduced stress gradient between NP and AF in the normal state could potentially mitigate the risk of annular injuries under extreme loading scenarios such as hyperflexion or whiplash [64]. As the simulation progressed, the anteroposterior fluid pressure gradient was also more rounded in cervical discs, which may reflect a more uniform pressurization response across the cervical IVD regions [47]. The fluid flux pattern was unique as it shifted from dominating the AF transport pathway immediately after the compressive ramp phase to the CEP pathway as equilibrium was approached. This difference, though negligible at equilibrium, suggests that convection through the CEP may be more restricted during the transient loading phase. Such phenomena could play a role in nutrient exchange stability and maintenance of the internal disc pressure during dynamic loading [57, 65]. However, investigation into the anisotropic permeability of the AF is needed to more fully elucidate the regional fluid transport dynamics in cervical discs.

While this study provides insights into the regional biomechanics of cervical discs, several limitations must be acknowledged. Inclusion of only three male donors of an older age range limits the generalizability of the reported biomechanical properties. Similarly, the effects of disc level were not evident in this dataset due to the limited number of discs analyzed. Other studies have demonstrated significant variations in bone mineral density across different regions and levels of the cervical spine [61, 66]. Therefore, it is possible that cervical IVD tissue also exhibits variability with disc level that the present study was unable to capture. Additionally, while no differences were observed between the biphasic properties of Thompson grade II and III specimens (see Online Resource 1, Supplemental Fig. 3), inclusion of grade III specimens with mild degeneration could have impacts of increased aggregate modulus and decreased permeability associated with lower water content [22, 63]. For these reasons, the present study should be regarded as pilot in nature. Permeability measurements exhibited high variability and lacked significant differentiation between IVD regions. Consistent with prior studies, poor sensitivity of this parameter may stem from issues related to the interdigitation of cartilage within the porous plate of the testing chamber [25, 63, 67]. This study also focused on axial compressive properties under confined compression conditions, which ideally causes collagen fibers to experience negligible tension. Consequently, the influence of collagen content on biphasic properties was not considered. However, the structural characteristics of collagen, particularly its degree of three-dimensional organization, may impact non-linear biphasic compressive behaviors, though this is usually only applicable for large creep strains (> 15%) [63]. While this study examined the axial compressive properties of cervical disc tissues, the cervical spine has more kinematic freedom than other spine sections. Future studies should expand upon these findings by investigating dynamic compression and tensile properties to better understand *in vivo* loading conditions. Finally, regarding the finite element model, we employed a simplified geometry to illustrate the likely influence of measured biphasic mechanical properties on matrix stress and fluid pressure within the IVD. Future studies are needed to validate these predictions in anatomically intact discs and to account for subject- and disc-specific morphological variability. In terms of biofidelity, the biphasic material model did not incorporate fiber reinforcement, which may be necessary to capture the tension–compression nonlinearity behavior of cartilaginous tissues [68, 69].

In conclusion, cervical intervertebral discs exhibit unique biomechanical adaptations that support their role in facilitating head mobility. Uniquely, the apparent homogeneity in NP and AF swelling pressure and aggregate modulus properties in cervical discs suggest a role in providing greater freedom of kinematic bending and stabilization of multidirectional mechanical loads. Elevated values of swelling pressure and aggregate modulus in the CEP confirm its importance as a mechanical barrier throughout the spine. Future research should focus on characterizing cervical discs’ dynamic and tensile properties and exploring how these adaptations change with aging or degeneration.

## Supplementary Information

Below is the link to the electronic supplementary material.

Supplementary file1 (PDF 980 kb)
