# Supplementary material for: Characterizing the Baseline Regional Biphasic Mechanical Properties of Cervical Intervertebral Discs
Source: Ann Biomed Eng. 2025 May 21;53(9):2333–45. doi: 10.1007/s10439-025-03759-2 (PMC12391200; doi:10.1007/s10439-025-03759-2)
Supplement: Supplementary file 1 — Supplementary file1 (PDF 980 kb) [file 10439_2025_3759_MOESM1_ESM.pdf]

Online Resource 1

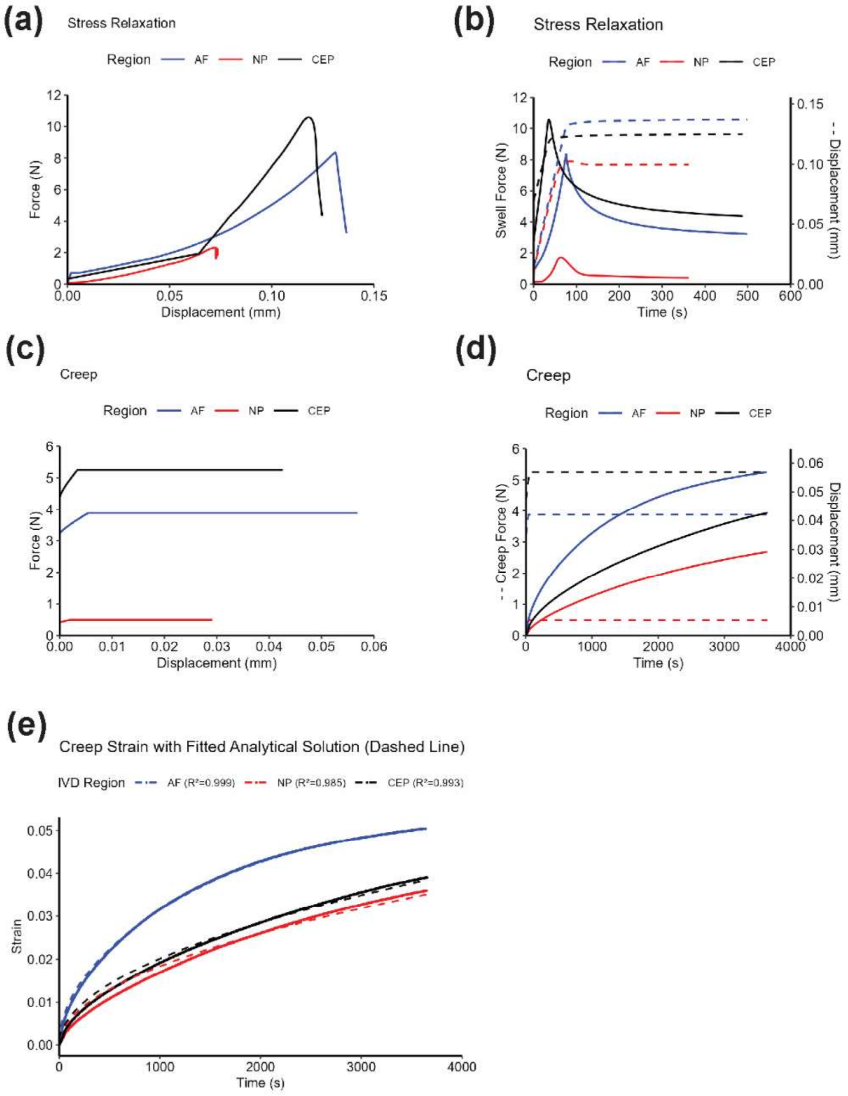


**Supplemental Figure 1:** Experimental traces for representative samples in each IVD region, including (a) force vs. displacement during stress relaxation, (b) force and displacement vs. time during stress relaxation, (c) force vs. displacement during creep loading, (d) force and displacement vs. time during creep loading, (e) linear biphasic curve-fitting of creep strain vs. time. The minimum $R^2$ from curve fitting was 0.89 and the average was 0.96.

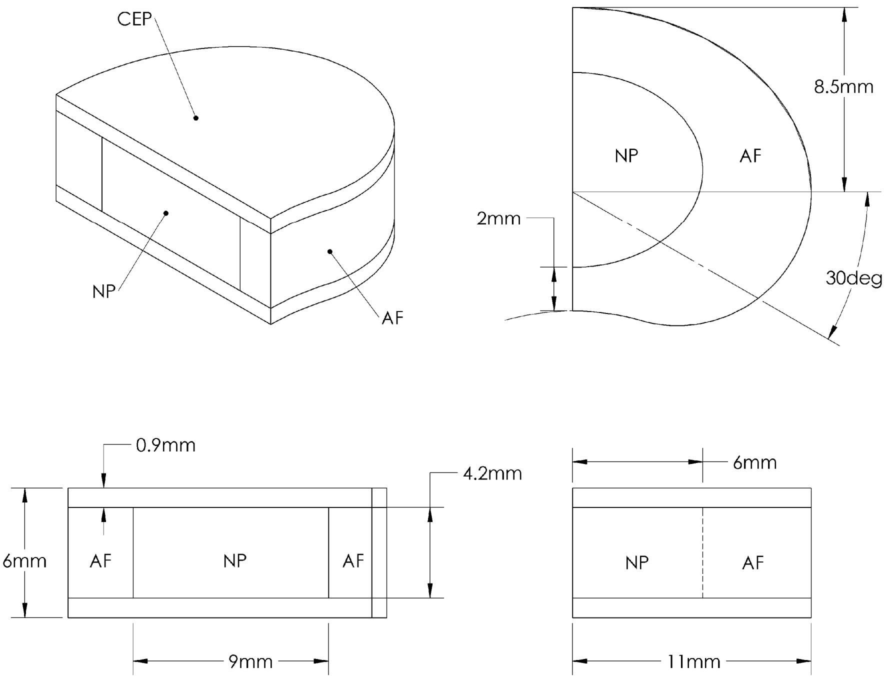


**Supplemental Figure 2:** Finite element model geometry specification, based on a C6-C7 disc. Only one half of the disc is modeled, because symmetry is assumed in the model solution. The transverse plane profile (upper and lower right-side panels) is estimated from a dissection image included in Figure 1 of a related study quantifying cervical disc fixed charge density[1].

1. Buchweitz, N., Y. Sun, S. Cisewski Porto, S. Wang, C. Reitman, H. Yao, and Y. Wu. Characterizing Baseline Fixed Charge Density in Human Cervical Intervertebral Discs. *J Biomech* (2025).

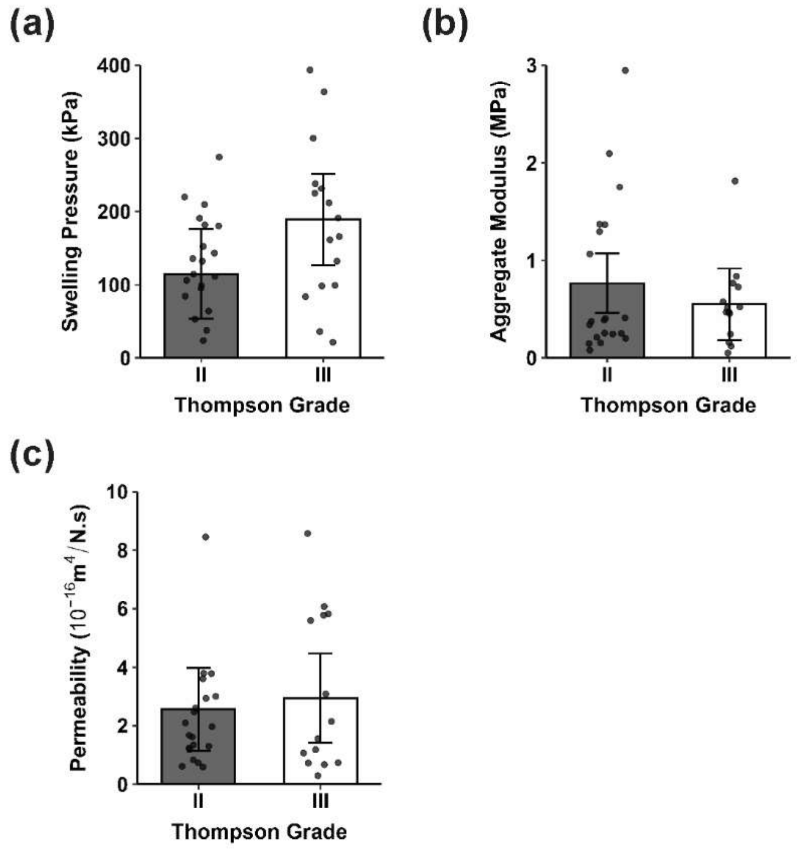


**Supplemental Figure 3:** Comparison of mechanical properties by Thompson grade, including (a) swelling pressure, (b) aggregate modulus, and (c) permeability. Due to unequal variances between grades, Welch's unpaired two-sample t-test was conducted at a significance threshold of $p < 0.05$. No statistical differences were detected between grades II and III for any of the measured properties. Sample sizes were unbalanced between grade and IVD region and consequently were too small for comparison of grades within each region.
